# Moderate-to-severe chronic spontaneous urticaria is associated with increased risk of immune-mediated diseases: A population-based study

**DOI:** 10.1371/journal.pone.0358883

**Published:** 2026-09-23

**Authors:** Kimberly Smart, Andrea Ure, Nicole Johnsen, Shaliz Aflatooni, Julian A. Cortes, Gabriela Palma, Yvonne Nong, Andrea J. Borba, Paul C. Levins, April W. Armstrong

**Affiliations:** 1 Keck School of Medicine, University of Southern California, Los Angeles, California, United States of America; 2 University of California, Davis School of Medicine, Sacramento, California, United States of America; 3 David Geffen School of Medicine at the University of California, Los Angeles, California, United States of America; 4 USF Morsani College of Medicine, Tampa, Florida, United States of America; 5 University of California, San Diego School of Medicine, San Diego, California, United States of America; 6 Donald and Barbara Zucker School of Medicine at Hofstra/Northwell, Uniondale, New York, United States of America; 7 Division of Dermatology, Department of Medicine, David Geffen School of Medicine at the University of California, Los Angeles, California, United States of America; Sungkyunkwan University - Suwon Campus: Sungkyunkwan University - Natural Sciences Campus, KOREA, REPUBLIC OF

## Abstract

Chronic spontaneous urticaria (CSU) is characterized by the idiopathic appearance of wheals and/or angioedema lasting more than six weeks. There is a critical need to evaluate whether the severity of CSU influences the risk of future immune-mediated comorbidities. We aim to evaluate if patients with moderate-to-severe chronic spontaneous urticaria (CSU) have an increased risk of developing immune-mediated comorbidities compared with matched patients with mild CSU. We conducted a retrospective cohort study using the TriNetX US Collaborative Network database from 2014–2026. We propensity matched patients with ≥2 ICD-10 diagnoses of idiopathic urticaria treated with omalizumab, dupilumab, or cyclosporine (moderate-to-severe disease) to age-, sex-, race-, ethnicity-, comorbidity-, and family history-matched counterparts who were never treated with biologics or cyclosporine (mild disease). After matching, 16,413 patients were included in the moderate-to-severe group, and 16,413 patients were included in the mild group. Patients with moderate-to-severe CSU had a significantly increased risk of developing rheumatoid arthritis OR=1.34 (95% CI: 1.12 to 1.60, p = 0.001) and Sjögren’s syndrome OR=1.04 (1.63–2.56, p < 0.0001), after adjusting for clinical factors and multiple hypothesis testing (Bonferroni’s alpha ≤0.00333). Moderate-to-severe CSU is associated with an increased risk of rheumatoid arthritis and Sjögren’s syndrome compared to mild CSU.

## Introduction

Chronic spontaneous urticaria (CSU) affects approximately 1% of individuals and is most prevalent in females aged 30–50 years old, highlighting the need to further investigate the immune-mediated comorbidities associated with CSU [1–3]. Approximately 10.5% to 28% of individuals with CSU are estimated to have at least one autoimmune comorbidity [3–6]. To our knowledge, no prior studies have analyzed whether the risk of developing these immune-mediated conditions differs based on CSU disease severity. Therefore, while a diagnosis of CSU has been associated with an increased risk of autoimmune conditions, there is a critical need to evaluate whether the severity of CSU influences the likelihood of developing immune-mediated comorbidities [4,7–9].

The severity of CSU can be considered as two groups: mild and moderate-to-severe. Those with mild disease are typically controlled with second generation antihistamines [10]. Those with more moderate-to-severe disease typically need additional therapies. These patients then can be considered candidates for treatment with omalizumab, dupilumab, remibrutinib, and cyclosporine [11]. This study aims to elucidate whether patients with treatment-defined moderate-to-severe CSU are at greater risk for developing immune-mediated conditions compared to patients with milder disease.

## Materials and methods

### Data source

We conducted a retrospective cohort study using the TriNetX US Collaborative Network database from 2014 to 2026. Data was captured and analyzed on May 24, 2026. All data were de-identified, and this study was considered exempt from review by the University of California, Los Angeles Institutional Review Board.

### Study population

The moderate-to-severe disease cohort was defined as patients ≥18 years old with at least 2 ICD-10 diagnoses of idiopathic urticaria (L50.1, L50.8, L50.9) six weeks apart who were treated with omalizumab, dupilumab, or cyclosporine. These treatments were chosen as they are the most commonly prescribed medications for patients who fail second-generation antihistamines [10]. Our mild disease cohort was defined as patients ≥18 years old with at least 2 ICD-10 diagnoses of idiopathic urticaria six weeks apart who were never treated with biologics or cyclosporine. Patients with a diagnosis of atopic dermatitis (AD) were excluded from both cohorts to avoid confounding as patients may be treated with dupilumab for AD. ICD-10 and RxNorm codes used are listed in Table 1.

**Table 1:** ICD-10 Codes and RxNorm Codes Used For Cohort Creation and Propensity Score Matching.

Treatment/ Diagnosis	RxNorm/ ICD-10 Code
Omalizumab	302379
Dupilumab	1876376
Cyclosporine	3008
Idiopathic Urticaria	L50.1, L50.8, L50.9
Atopic Dermatitis	L20, L20.8, L20.81-L20.84, L20.89, L20.9
Vitiligo	L80
Sjogren’s Syndrome	M35.0
Autoimmune thyroiditis	E06.3
Pernicious anemia	D51.0
Systemic lupus erythematosus	M32
Celiac disease	K90.0
Graves’ disease	E05.00
Scleroderma	M34
Fibromyalgia	M79.7
Crohn’s disease	K50
Ulcerative colitis	K51
Rheumatoid arthritis	M05, M06
Type 1 diabetes mellitus	E10
Psoriasis	L40, L40.0-L40.4, L40.8, L40.9
Psoriatic arthritis	L40.50-L40.54, L40.59
Asthma	J45
Insufficient health insurance coverage	Z59.71
Insufficient social insurance and welfare support	Z59.7
Persons with potential health hazards related to socioeconomic and psychosocial circumstances	Z55-Z65
Low income	Z59.6
Problems related to housing and economic circumstances	Z59
Tobacco Use (Smoking Status)	Z72
Obesity	E66
Family history of other diseases of the musculoskeletal system and connective tissue	Z82.69
Family history of arthritis	Z82.61
Family history of other endocrine, nutritional and metabolic diseases	Z83.4
Family history of diabetes mellitus	Z83.3
Family history of diseases of the blood and blood-forming organs and certain disorders involving the immune mechanism	Z83.2
Family history of other diseases of the digestive system	Z83.79

We propensity matched patients with moderate-to-severe disease to age-, sex-, race-, and ethnicity-matched counterparts with mild disease. Patients were also matched on comorbid asthma, obesity, tobacco use, family history of several autoimmune conditions, and socioeconomic factors related to insurance coverage and income. Propensity score matching was performed within the TriNetX platform using logistic regression to estimate the propensity score and 1:1 greedy nearest-neighbor matching with caliper width of 0.1 pooled standard deviations (SD). Standard mean differences (SMD) are reported in Table 2. After matching, 16,413 patients were included in the moderate-to-severe cohort, and 16,413 patients were included in the mild cohort. Baseline characteristics after matching are listed in Table 2.

**Table 2:** Baseline Characteristics After 1:1 Greedy Nearest Neighbor Propensity Score Matching.

	Before Matching						After Matching				
Demographics	Cohort	Mean + /- SD	Patients	% of Cohort	p value	Standard mean difference	Mean + /- SD	Patients	% of Cohort	p value	Standard mean difference
Age at Index	Moderate-to-Severe	44 + /- 18.6	16,415	100.0	<0.0001	0.4563	44 + /- 18.6	16,413	100.0	0.4869	0.0077
	Mild	34.4 + /- 23.1	145,135	100.0			44.1 + /- 18.9	16,414	100.0		
Female	Moderate-to-Severe		12,976	79.1	<0.0001	0.2190		12,974	79.0	0.7240	0.0039
	Mild		100,918	69.5				13,000	79.2		
Male	Moderate-to-Severe		3,427	20.9	<0.0001	0.2201		3,427	20.9	0.7442	0.0036
	Mild		44,169	30.4				3,403	20.7		
White	Moderate-to-Severe		11,591	70.6	<0.0001	0.1982		11,589	70.6	0.4891	0.0076
	Mild		88,918	61.3				11,646	71.0		
Asian	Moderate-to-Severe		724	4.4	<0.0001	0.1337		724	4.4	0.4475	0.0084
	Mild		10,999	7.8				696	4.2		
Black or African American	Moderate-to-Severe		2,116	12.9	<0.0001	0.0863		2,116	12.9	0.8951	0.0015
	Mild		23,102	15.9				2,108	12.8		
American Indian or Alaska Native	Moderate-to-Severe		90	0.5	0.9616	0.0004		90	0.5	0.5936	0.0059
	Mild		800	0.6				83	0.5		
Native Hawaiian or Other Pacific Islander	Moderate-to-Severe		45	0.3	<0.0001	0.0927		45	0.3	0.8309	0.0024
	Mild		1,474	1.0				43	0.3		
Other Race	Moderate-to-Severe		613	3.7	<0.0001	0.0598		613	3.7	0.6605	0.0048
	Mild		7,188	5.0				598	3.6		
Unknown Race	Moderate-to-Severe		1,236	7.5	<0.0001	0.0435		1,236	7.5	0.95	0.0007
	Mild		12,654	9.7				1,239	7.5		
Hispanic or Latino	Moderate-to-Severe		1,255	7.6	<0.0001	0.1412		1,255	7.6	0.4026	0.0092
	Mild		17,156	11.8				1,215	7.4		
Not Hispanic or Latino	Moderate-to-Severe		11,818	72.0	<0.0001	0.0595		11,816	72.0	0.9510	0.0007
	Mild		100,562	69.3				11,811	72.0		
Unknown Ethnicity	Moderate-to-Severe		3,342	20.4	<0.0001	0.0370		3,342	20.4	0.5384	0.0068
	Mild		27,417	18.9				3,387	20.6		
Asthma	Moderate-to-Severe		5,342	32.5	<0.0001	0.2593		5,340	32.5	0.2934	0.0116
	Mild		30,690	21.1				5,251	32.0		
Obesity	Moderate-to-Severe		3,868	23.6	<0.0001	0.0885		3,868	23.6	0.3278	0.0108
	Mild		28,909	21.1				3,793	23.1		
Tobacco User	Moderate-to-Severe		511	3.1	0.9613	0.0004		511	3.1	0.0008	0.0371
	Mild		4,508	3.1				469	3.0		
Persons with potential health hazards related to socioeconomic and psychosocial circumstances	Moderate-to-Severe		831	5.1	0.0166	0.02		831	5.1	<0.0001	0.0445
	Mild		7,998	5.5				678	4.1		
Family history of diabetes mellitus	Moderate-to-Severe		610	3.7	<0.0001	0.0438		610	3.7	0.0008	0.0371
	Mild		4,253	2.9				500	3.0		
Family history of other endocrine, nutritional and metabolic diseases	Moderate-to-Severe		339	2.1	<0.0001	0.0348		338	2.1	0.0041	0.0317
	Mild		2,321	1.6				268	1.6		
Problems related to housing and economic circumstances	Moderate-to-Severe		211	1.3	0.5675	0.0047		211	1.3	<0.0001	0.0507
	Mild		1,790	1.2				127	0.8		
Family history of other diseases of the digestive system	Moderate-to-Severe		187	1.1	<0.0001	0.054		185	1.1	0.0102	0.0284
	Mild		919	0.6				139	0.8		
Family history of diseases of the blood and blood-forming organs and certain disorders involving the immune mechanism	Moderate-to-Severe		136	0.8	0.0003	0.028		136	0.8	0.0055	0.0307
	Mild		861	0.6				94	0.6		
Family history of arthritis	Moderate-to-Severe		124	0.8	<0.0001	0.0452		124	0.8	<0.0001	0.0457
	Mild		597	0.4				67	0.4		
Family history of other diseases of the musculoskeletal system and connective tissue	Moderate-to-Severe		70	0.4	0.0014	0.0241		70	0.4	0.0674	0.0202
	Mild		411	0.3				50	0.3		
Insufficient social insurance and welfare support	Moderate-to-Severe		18	0.1	0.79	0.0022		18	0.1	0.1304	0.0167
	Mild		170	0.1				10	0.1		
Insufficient health insurance coverage	Moderate-to-Severe		17	0.1	0.6761	0.0034		17	0.1	0.1778	0.0149
	Mild		135	0.1				10	0.1		
Low income	Moderate-to-Severe		16	0.1	0.7276	0.0029		16	0.1	0.2391	0.013
	Mild		155	0.1				10	0.1		

### Time period

The index date was defined as the second diagnosis of idiopathic urticaria in both groups. Patients with the outcomes of interest (immune-mediated comorbidities) prior to the index date were excluded. Patients were censored at the earliest diagnosis of the comorbidity of interest or at the end of the study period.

### Data analysis

Analysis was conducted using the TriNetX software. Odd ratios of developing each immune-mediated comorbidity at least 1 day after the index date (second diagnosis of idiopathic urticaria) were calculated. The incidence rates of immune-mediated comorbidities were calculated, and these rates were compared using the incidence rate ratio.

## Results

### Cohort demographics

The average age at diagnosis of CSU was 45.7 years in both groups. Our cohort was 77.6% female, 20.2% male, 72.0% non-Hispanic/Latino, 8.0% Hispanic/Latino, 20.0% Unknown Ethnicity, 70.3% White, 11.0% Black, 4.2% Asian, 0.4% American Indian/Alaska Native, 0.2% Native Hawaiian/Other Pacific Islander, 4.4% Other Race, and 9.4% Unknown Race.

### Odds of immune-mediated comorbidities

Patients with moderate-to-severe CSU had a statistically significant increased odds of developing rheumatoid arthritis OR=1.34 (95% CI: 1.12 to 1.60, p = 0.001) and Sjögren’s syndrome OR=1.04 (1.63–2.56, p < 0.0001), after adjusting for clinical factors and multiple hypothesis testing (Bonferroni’s alpha ≤0.00333).

**Fig 1:**
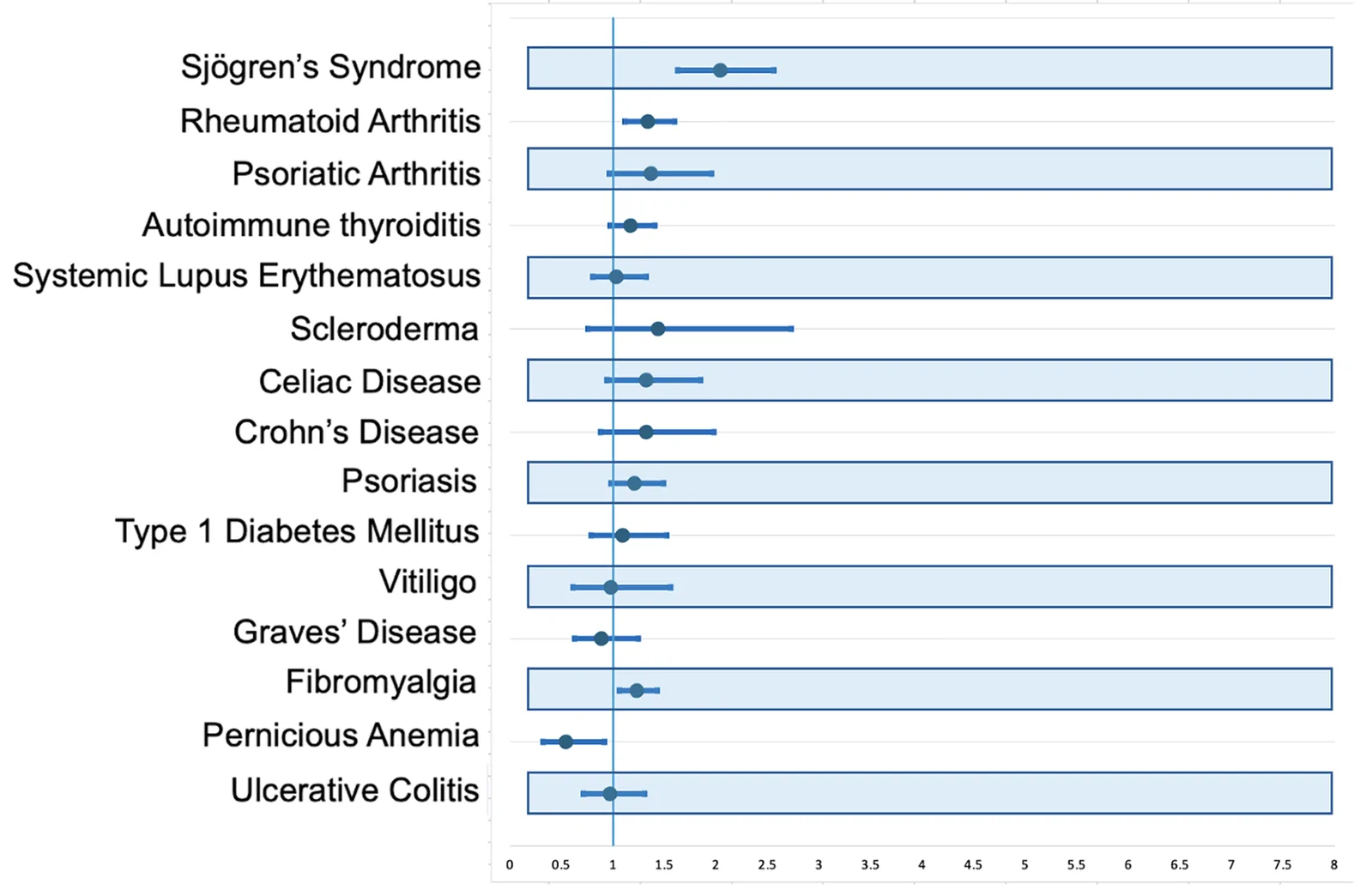
Forest plot showing odds ratios (ORs) and 95% confidence intervals for immune-mediated comorbidities among patients with chronic spontaneous urticaria (CSU). Odds ratios were adjusted for demographic and clinical covariates including age, sex, race/ethnicity, baseline comorbidities, and family history. Error bars represent 95% confidence intervals, and statistical significance was defined as p ≤ 0.00333.

There was no statistically significant difference in the odds of developing autoimmune thyroiditis OR=1.17, (95% CI: 0.97–1.41), vitiligo OR= 0.98 (95% CI: 0.61–1.56), pernicious anemia OR=0.54 (95% CI: 0.32–0.92), systemic lupus erythematosus (SLE) OR=1.03 (95% CI: 0.80–1.32), celiac disease OR=1.32 (95% CI: 0.94–1.85), Graves’ disease OR=0.89 (95% CI: 0.63–1.25), Crohn’s disease OR=1.32 (95% CI: 0.88–1.98), ulcerative colitis (UC) OR=0.97 (95% CI: 0.71–1.31), scleroderma OR= 1.44 (95% CI: 0.76–2.73), fibromyalgia OR=1.23 (95% CI: 1.06–1.43, type 1 diabetes mellitus OR= 1.09 (95% CI: 0.79–1.52), psoriatic arthritis OR=1.37 (95% CI:0.96–1.96), and psoriasis OR= 1.21 (95% CI: 0.98,1.49). Odds ratios are depicted in Fig 1.

### Incidence rates of immune-mediated comorbidities

The incidence rate for rheumatoid arthritis in the moderate-to-severe CSU cohort was 1.52 cases per 1000 person-years, while the incidence rate for rheumatoid arthritis in the mild CSU cohort was 1.14 cases per 1000 person-years. The incidence rate ratio for rheumatoid arthritis was 1.33 (95% CI: 1.12 to 1.60, p = 0.0014). The incidence rate for Sjögren’s syndrome in the moderate-to-severe CSU cohort was 1.20 cases per 1000 person-years, while the incidence rate for Sjögren’s syndrome in the mild CSU cohort was 0.59 cases per 1000 person-years. The incidence rate ratio for Sjögren’s syndrome was 2.03 (95% CI: 1.62 to 2.53, p < 0.0001).

### Cumulative incidence of immune-mediated comorbidities

**Fig 2:**
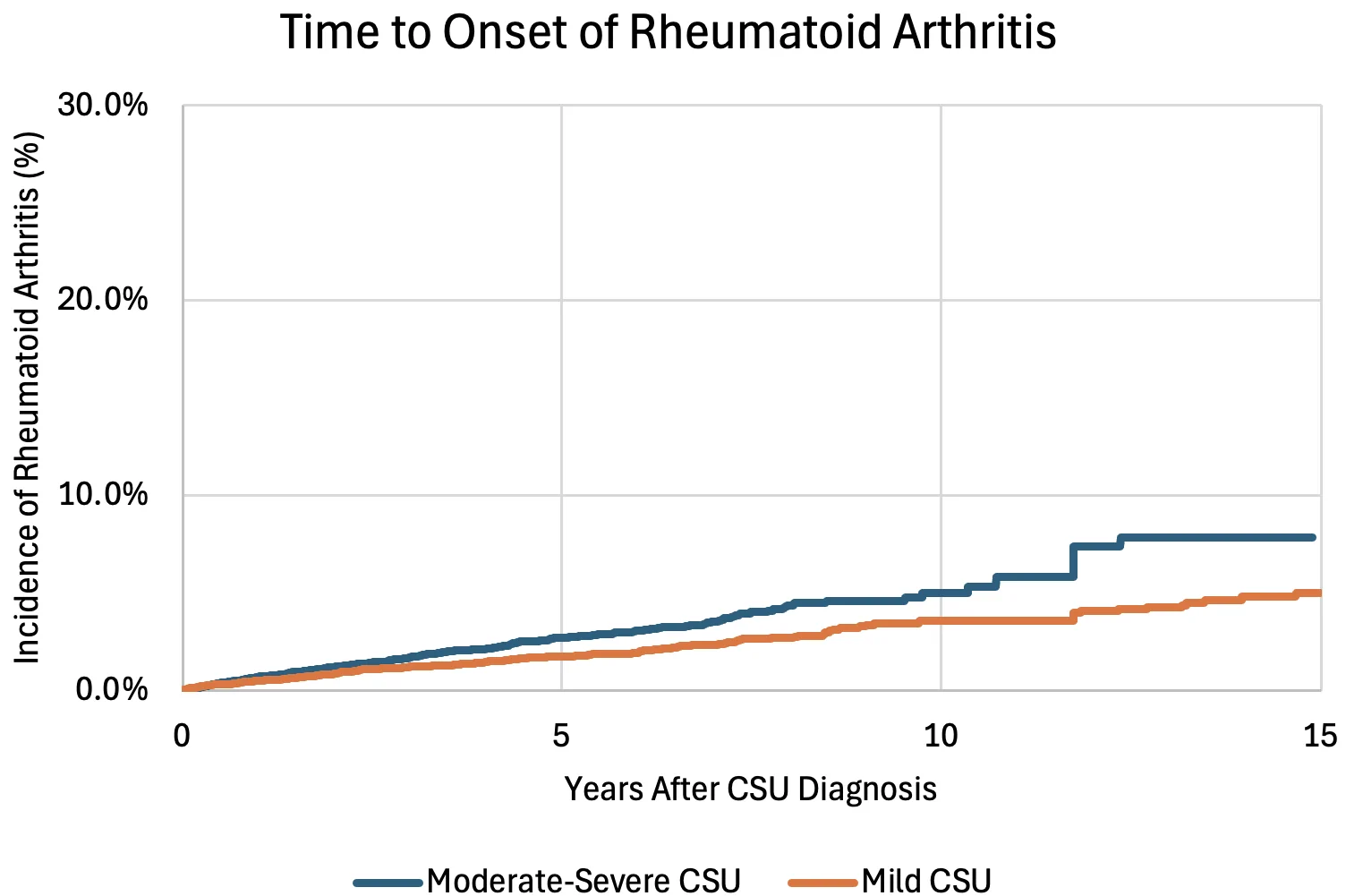
Kaplan-Meier curve demonstrating time to development of rheumatoid arthritis among patients with moderate-to-severe CSU compared with mild CSU. Time-to-event analyses were performed using Cox proportional hazards modeling adjusted for relevant demographic and clinical covariates, including age, sex, race/ethnicity, baseline comorbidities, and family history.

**Fig 3:**
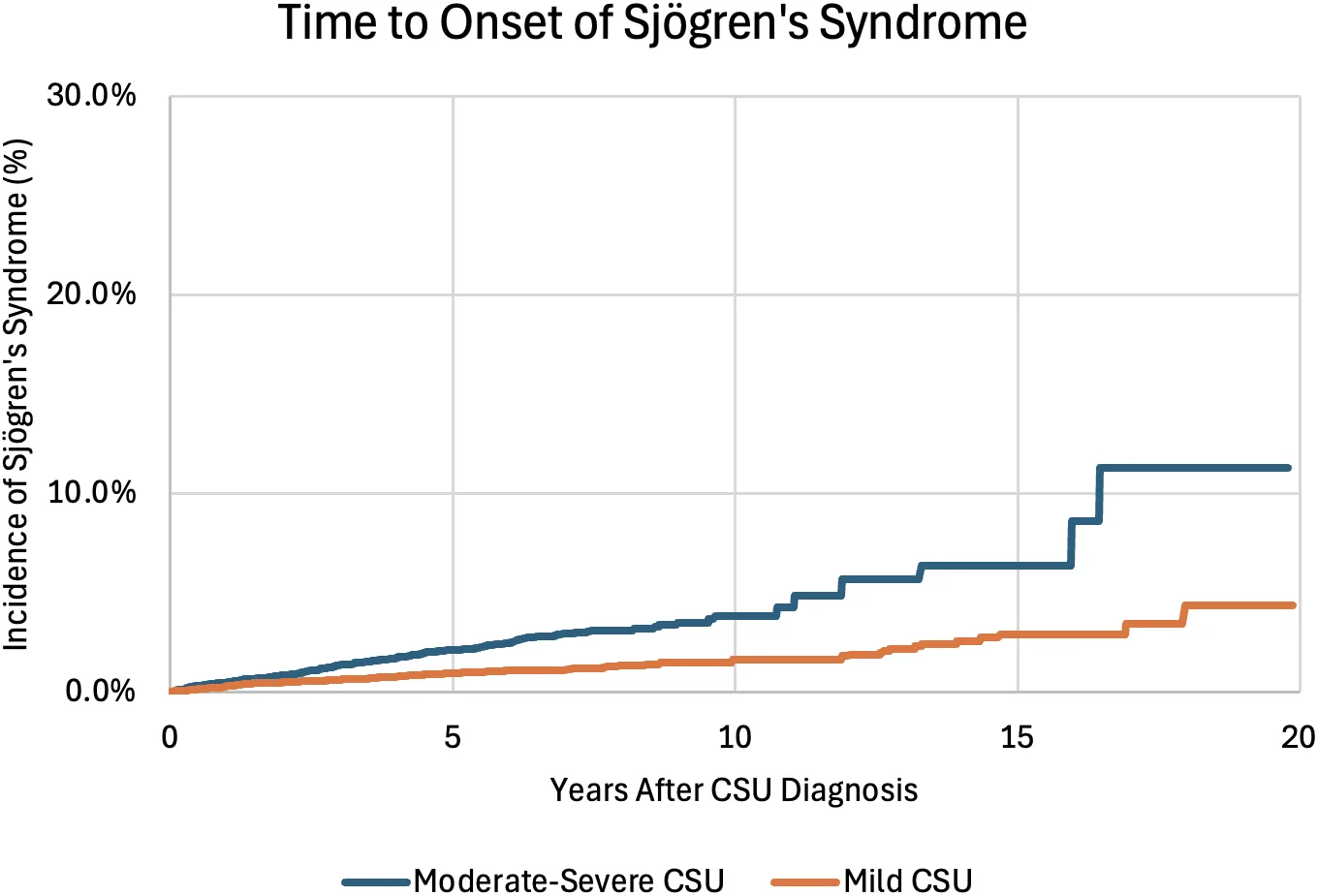
Kaplan-Meier curve demonstrating time to development of Sjögren’s syndrome among patients with moderate-to-severe CSU compared with mild CSU. Time-to-event analyses were performed using Cox proportional hazards modeling adjusted for relevant demographic and clinical covariates, including age, sex, race/ethnicity, baseline comorbidities, and family history.

The cumulative incidence of rheumatoid arthritis was 285 patients (1.8%) in the moderate-to-severe CSU cohort compared to 217 patients (1.4%) in the mild CSU cohort (p = 0.0013). The cumulative incidence of Sjögren’s syndrome was 226 patients (1.4%) in the moderate-to-severe CSU cohort and 115 patients (0.7%) in the mild CSU cohort (p < 0.0001). Kaplan Meier estimates depicting time to event analyses for rheumatoid arthritis and Sjögren’s syndrome are shown in Figs 2 and 3.

### Absolute risk difference of immune-mediated comorbidities

**Fig 4:**
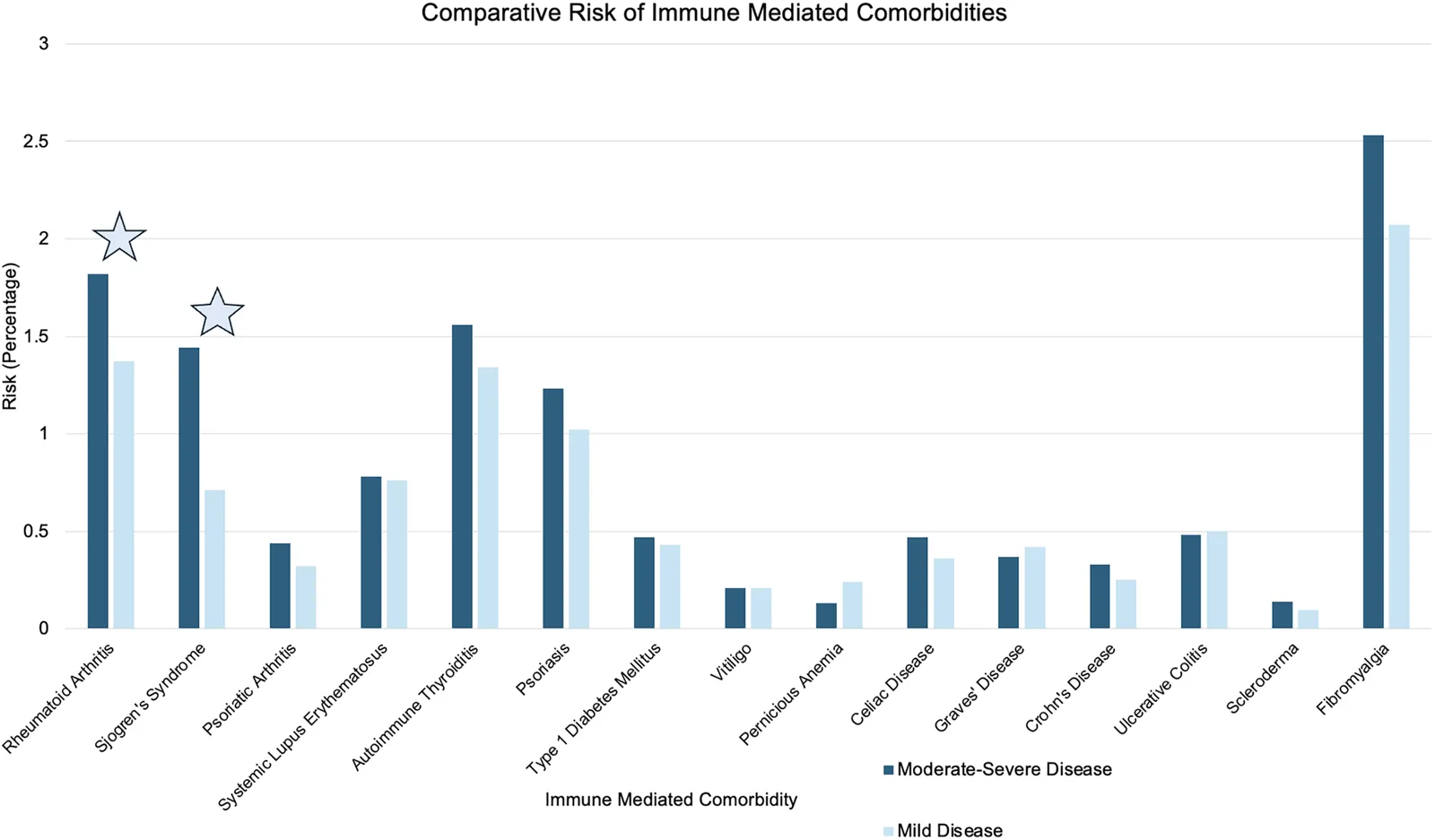
Absolute risk of immune-mediated comorbidities among age-, sex-, race/ethnicity-, baseline comorbidity-, and family history-propensity score-matched patients with moderate-to-severe CSU and mild CSU. For each outcome, the number of patients who developed the immune-mediated comorbidity was calculated among patients at risk within each matched cohort and compared between groups. Absolute risk is presented as the proportion of patients developing each outcome during the study period. Statistical significance was adjusted using Bonferroni’s alpha ≤0.00333.

The absolute risk difference of developing these immune-mediated conditions in patients with moderate-to-severe CSU compared to those with mild CSU was calculated. Patients with moderate-to-severe CSU had a 0.5% increased absolute risk of developing rheumatoid arthritis (p = 0.0013) and a 0.7% increased absolute risk of developing Sjögren’s syndrome (p < 0.0001), as shown in Fig 4.

## Discussion

This is among the initial efforts to characterize CSU-associated immune-mediated conditions based on treatment-defined disease severity. More severe CSU disease correlates with an increased risk of new-onset rheumatoid arthritis and Sjögren’s syndrome. While CSU has been associated with various autoimmune conditions in the literature, our findings corroborate and expand upon the existing literature by demonstrating that CSU disease severity correlates with the risk of some, but not all, new-onset immune-mediated comorbidities [2,4,5,10,12]. Our findings are based on robust analysis using multiplicity adjustment. This knowledge is important to anticipate the prognosis and disease course of CSU and optimize care management for these patients.

The pathogenesis of CSU may be linked to the development of rheumatoid arthritis through shared autoimmune pathways, genetic susceptibility, and the presence of autoantibodies. Patients with CSU have an increased prevalence of rheumatoid factor and antinuclear antibody, possibly indicating a shared autoimmune pathogenesis between rheumatoid arthritis and CSU [4]. A prior genome-wide association study (GWAS) revealed that the *PTPN22* gene is associated with increased risk for both rheumatoid arthritis and CSU [12]. Another study using polygenic risk score (PRS) found a genetic relationship between CSU and rheumatoid arthritis [13]. Additionally, a mendelian randomization analysis also showed a genetic association between rheumatoid arthritis and CSU, but not between CSU and SLE, UC, or Crohn’s disease [14]. These shared genetic pathways observed in prior literature potentially further explain our finding that more severe CSU disease is associated with an increased risk of rheumatoid arthritis.

Prior research on Sjögren’s syndrome is much more limited. The authors hypothesize that this association between more severe CSU disease and Sjögren’s syndrome may be due to shared immune-mediated pathways; however, further research is needed to elucidate this mechanism.

Given that we did not find a statistically significant difference in the odds of new onset autoimmune thyroid disease (AITD), we suggest that the type IIb CSU subtype, rather than more severe CSU, is associated with increased risk for AITD. This hypothesis is driven by prior studies that showed CSU patients who had increased IgE-anti-TPO expression also had increased IgG-anti-TPO expression, a diagnostic indicator of AITD [15,16]. In this subset of CSU patients, IgE-anti-TPO antibodies, among others, bind to and degranulate mast cells, resulting in recurrent hives [17]. Further studies are needed to investigate this possible association.

Importantly, the risk of several other autoimmune conditions, namely vitiligo, pernicious anemia, systemic lupus erythematosus, celiac disease, Graves’ disease, Crohn’s disease, ulcerative colitis, scleroderma, fibromyalgia, type 1 diabetes mellitus, psoriatic arthritis, and psoriasis, was not significantly increased in patients with more severe CSU disease. Therefore, we suggest that these immune-mediated conditions may not share the same pathway as CSU. However, further research is necessary to elucidate these relationships.

In our study, there is a potential for misclassification due to the nature of utilizing large database electronic health record (EHR) data and the use of treatment type as a proxy for disease severity. Medication indication is not available within this database, resulting in potential misclassification and off-label use. Another limitation is the potential for detection bias, as patients with more severe CSU may have more frequent healthcare visits, potentially leading to earlier diagnosis of comorbidities. Because validated CSU activity measures were unavailable in TriNetX, treatment escalation was used as a proxy for moderate-to-severe disease. This treatment-defined severity measure may be influenced by access, insurance coverage, prescribing patterns, and healthcare utilization, creating potential misclassification. Thus, findings should be interpreted as associations with treatment-defined moderate-to-severe CSU rather than direct clinical severity.

Nevertheless, our findings emphasize the importance of comprehensive interdisciplinary management of patients with CSU, especially those presenting with more severe disease. These patients will need to be followed closely for the development of comorbid immune-mediated diseases, particularly rheumatoid arthritis and Sjögren’s syndrome.

## References

[1] Pourali SP, Gutierrez Y, Kucharik AH, Rajkumar JR, Jones ME, Armstrong AW. Chronic spontaneous urticaria in the United States: Patient characteristics and treatment patterns. Int J Dermatol. 2022;61(8):e308–9. doi: 10.1111/ijd.15835 34370872

[2] Sánchez-Borges M, Ansotegui IJ, Baiardini I, Bernstein J, Canonica GW, Ebisawa M, et al. The challenges of chronic urticaria part 1: Epidemiology, immunopathogenesis, comorbidities, quality of life, and management. World Allergy Organ J. 2021;14(6):100533. doi: 10.1016/j.waojou.2021.100533 34221215 PMC8233382

[3] Kolkhir P, Bonnekoh H, Metz M, Maurer M. Chronic spontaneous urticaria: a review. JAMA. 2024;332(17):1464–77. doi: 10.1001/jama.2024.15568 39325444

[4] Confino-Cohen R, Chodick G, Shalev V, Leshno M, Kimhi O, Goldberg A. Chronic urticaria and autoimmunity: Associations found in a large population study. J Allergy Clin Immunol. 2012;129(5):1307–13. doi: 10.1016/j.jaci.2012.01.043 22336078

[5] Kolkhir P, Altrichter S, Asero R, Daschner A, Ferrer M, Giménez-Arnau A, et al. Autoimmune diseases are linked to type IIb autoimmune chronic spontaneous urticaria. Allergy Asthma Immunol Res. 2021;13(4):545–59. doi: 10.4168/aair.2021.13.4.545 34212543 PMC8255350

[6] Buttgereit T, Vera C, Aulenbacher F, Church MK, Hawro T, Asero R, et al. Patients with chronic spontaneous urticaria who have wheals, angioedema, or both, differ demographically, clinically, and in response to treatment-results from CURE. J Allergy Clin Immunol Pract. 2023;11(11):3515–3525.e4. doi: 10.1016/j.jaip.2023.08.020 37604426

[7] Patel D, Pathak N, Alani O, Singal A, Lipner SR. Autoimmune associations with chronic spontaneous urticaria: A retrospective cohort analysis. JAAD Int. 2025;21:44–6. doi: 10.1016/j.jdin.2025.05.002 40672397 PMC12266570

[8] Pourali SP, Kohn AH, Jones ME, Armstrong AW. Chronic spontaneous urticaria: A 16-year analysis of pediatric patient demographics, treatment patterns, and comorbidities. Dermatol Online J. 2021;27(8). doi: 10.5070/D327854723 34755971

[9] Kaplan A, Lebwohl M, Giménez-Arnau AM, Hide M, Armstrong AW, Maurer M. Chronic spontaneous urticaria: Focus on pathophysiology to unlock treatment advances. Allergy. 2023;78(2):389–401. doi: 10.1111/all.15603 36448493

[10] Lang DM, Sheikh J, Joshi S, Bernstein JA. Endotypes, phenotypes, and biomarkers in chronic spontaneous urticaria: Evolving toward personalized medicine. Ann Allergy Asthma Immunol. 2025;134(4):408–17.e3. doi: 10.1016/j.anai.2024.10.026 39490777

[11] Kolkhir P, Fok JS, Kocatürk E, Li PH, Okas TL, Marcelino J, et al. Update on the treatment of chronic spontaneous urticaria. Drugs. 2025;85(4):475–86. doi: 10.1007/s40265-025-02170-4 40074986 PMC11946961

[12] Zhang L, Qiu L, Wu J, Qi Y, Gao X, He C, et al. GWAS of chronic spontaneous urticaria reveals genetic overlap with autoimmune diseases, not atopic diseases. J Invest Dermatol. 2023;143(1):67–77.e15. doi: 10.1016/j.jid.2022.07.012 35933036

[13] Chang D, Hammer C, Holweg CTJ, Selvaraj S, Rathore N, McCarthy MI, et al. A genome-wide association study of chronic spontaneous urticaria risk and heterogeneity. J Allergy Clin Immunol. 2023;151(5):1351–6. doi: 10.1016/j.jaci.2022.10.019 36343773

[14] Yang M, Su Y, Xu K, Wen P, Zhang B, Guo J, et al. Common autoimmune diseases and urticaria: The causal relationship from a bidirectional two-sample mendelian randomization study. Front Immunol. 2023;14:1280135. doi: 10.3389/fimmu.2023.1280135 38022623 PMC10652397

[15] Asero R, Ferrer M, Kocaturk E, Maurer M. Chronic spontaneous urticaria: The role and relevance of autoreactivity, autoimmunity, and autoallergy. J Allergy Clin Immunol Pract. 2023;11(8):2302–8. doi: 10.1016/j.jaip.2023.02.022 36868473

[16] Xie L-D, Gao Y, Li M-R, Lu G-Z, Guo X-H. Distribution of immunoglobulin G subclasses of anti-thyroid peroxidase antibody in sera from patients with Hashimoto’s thyroiditis with different thyroid functional status. Clin Exp Immunol. 2008;154(2):172–6. doi: 10.1111/j.1365-2249.2008.03756.x 18778360 PMC2612718

[17] Altrichter S, Peter HJ, Pisarevskaja D, Metz M, Martus P, Maurer M. IgE mediated autoallergy against thyroid peroxidase – A novel pathomechanism of chronic spontaneous urticaria? PLoS ONE. 2011;6(4):e14794. doi: 10.1371/journal.pone.0014794 21532759 PMC3075251

